# Delayed Hypoglossal Nerve Injury After Traumatic Skull Base Fracture: A Case Report and Literature Review

**DOI:** 10.1002/ccr3.70053

**Published:** 2025-03-23

**Authors:** Malik S. Obeidallah, Max Fleisher, Peter Harris, Khashayar Mozaffari, Michael Rosner

**Affiliations:** ^1^ Department of Neurosurgery George Washington University Washington DC USA

**Keywords:** delayed, hypoglossal, palsy, trauma

## Abstract

Our case demonstrates that delayed hypoglossal palsy secondary to trauma can be resolved with conservative, non‐operative management with a team‐based approach.

## 1 Introduction

The hypoglossal nerve represents the twelfth and final cranial nerve. Since it concludes most cranial nerve exams, and isolated injuries to this nerve are relatively rare, it is often neglected. However, it plays an integral role in chewing, swallowing, oral hygiene, and speech articulation. The hypoglossal nerve can be divided into cisternal, canal, descending, horizontal, and ascending, from proximal to distal. The muscular branch originates in the hypoglossal nucleus of the medulla, passes through the hypoglossal canal of the skull base, and supplies the intrinsic and extrinsic tongue muscles. Other branches originate from C1 to C3 nerve roots and reconvene via the ansa cervicalis [1].

Hypoglossal nerve injuries can be classified as acute or delayed in onset. They can be further categorized by their etiology, which includes, but is not limited to, vascular causes, trauma, neoplastic, iatrogenic injury, infectious, environmental (toxic), and autoimmune.^1^ Traumatic fractures of the occipital condyle frequently involve the hypoglossal canal, and, by association, the muscular branch of the hypoglossal nerve. The fracture can cause an acute hypoglossal palsy, or, in the event of callus formation that occludes the canal, a delayed‐onset twelfth nerve palsy [2, 3, 4].

This case represents a rare instance of a delayed hypoglossal nerve (HGN) injury arising 3 months after an occipital condyle fracture (OCF) caused by a motor vehicle accident. Management of OCF often fails to prevent or relieve HNP over time, with chronic persistence of tongue deviation and atrophy, dysphagia, and dysarthria. To date, there are only 11 cases of delayed isolated HNP after skull base trauma in the literature. Of these cases, only three reported complete resolution [2, 5, 6]. This case illustrates successful non‐operative management of delayed HNP, a documented but rare complication of skull base fractures adjacent to the hypoglossal canal.

## 2 Case History and Examination

**FIGURE 1:**
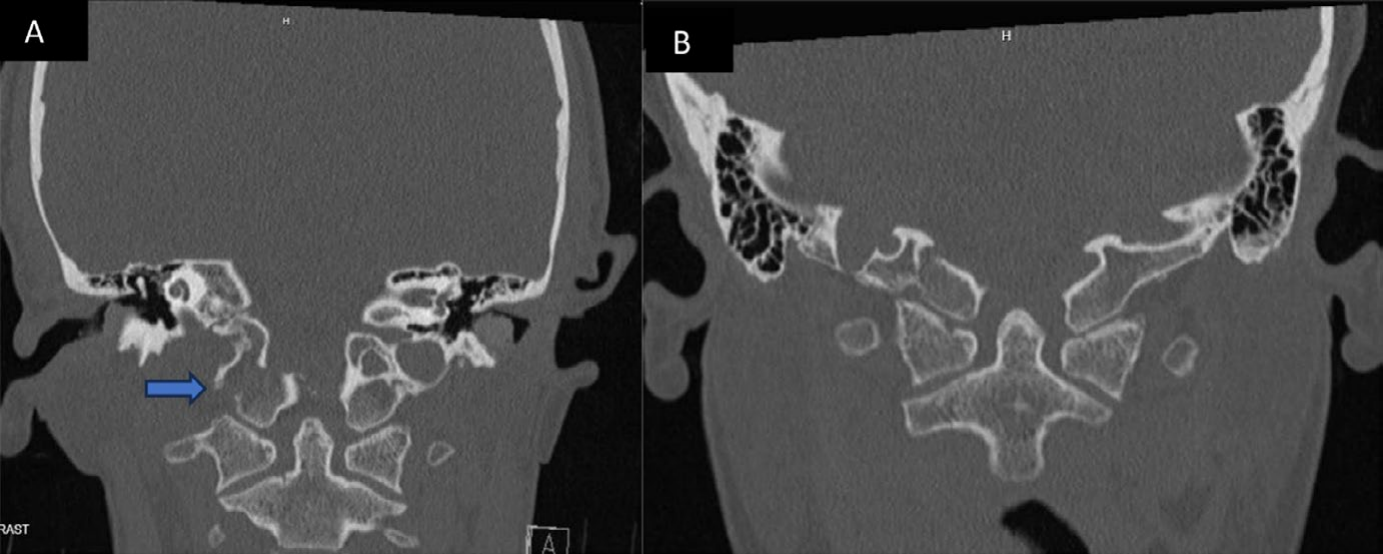
A and B non‐contrast CT maxillofacial (coronal view) showing initial avulsion type fracture through right occipital condyle, and 3 months follow up CT in a similar plane showing healed fracture.

**FIGURE 2:**
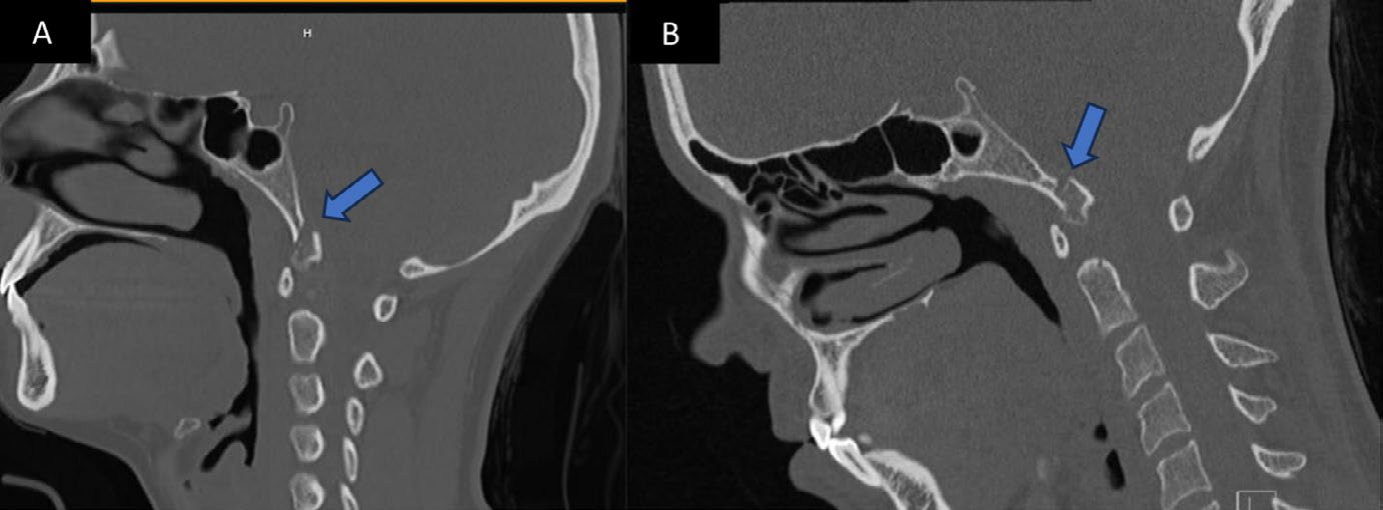
A and B non‐contrast CT maxillofacial (sagittal view) showing initial avulsion type fracture through right occipital condyle, and 3 months follow up CT in a similar plane showing callus formation.

**FIGURE 3:**
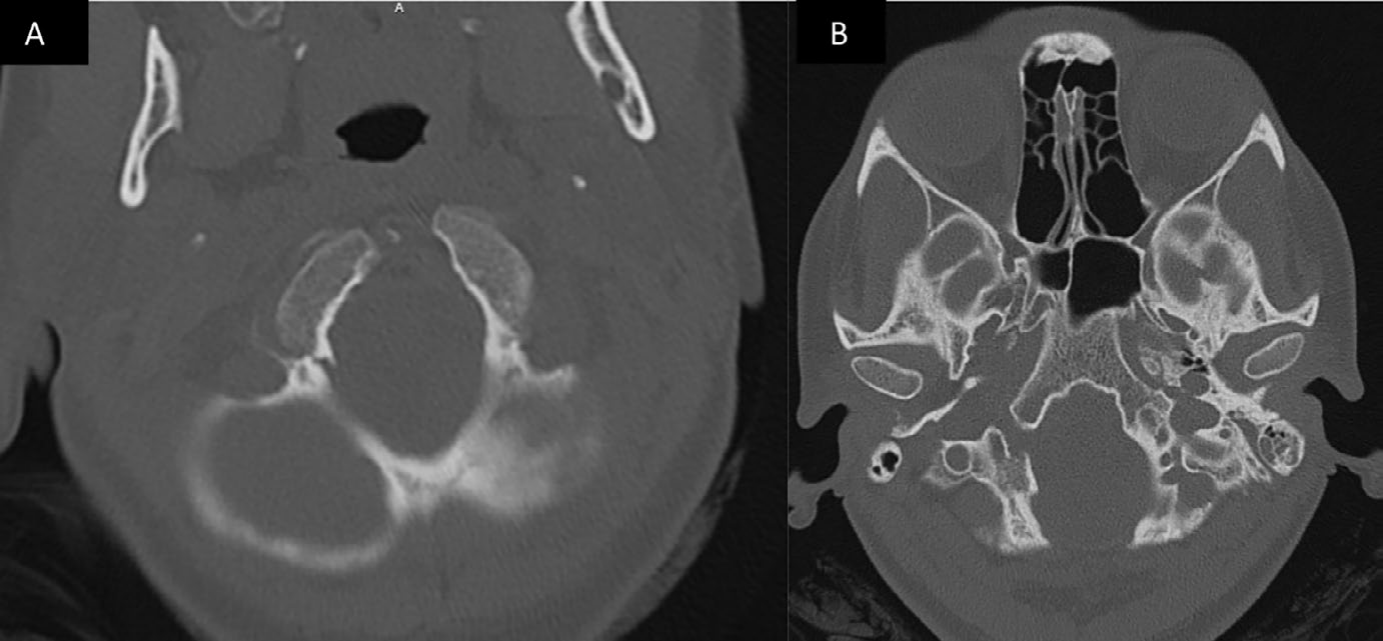
A and B non‐contrast CT maxillofacial (axial view) showing initial avulsion type fracture through right occipital condyle, and 3 months follow up CT showing narrowing of the hypoglossal canal due to callus formation.

A 25‐year‐old female without significant past medical history presented to the ED following a car accident in which she was a restrained passenger. Imaging was performed in light of facial abrasions, neck pain, and tingling in both hands. Computerized tomography (CT) revealed an occipital condyle fracture, classified as Anderson and Montesano type III (Figures 1, 2, 3). Other injuries included fractures of the left thumb and radius, which were treated surgically. She was otherwise intact neurologically. A magnetic resonance image (MRI) of the cervical spine did not show any ligamentous disruption or cord compromise. After discussing various treatment options including surgical fixation, halo immobilization and Aspen collar, the patient elected to wear a collar for 8–10 weeks. At 2‐month follow up, she noted gradual improvement in her presenting symptoms, and after confirmation on X‐ray that the fracture had healed, her brace was discontinued.

**FIGURE 4:**
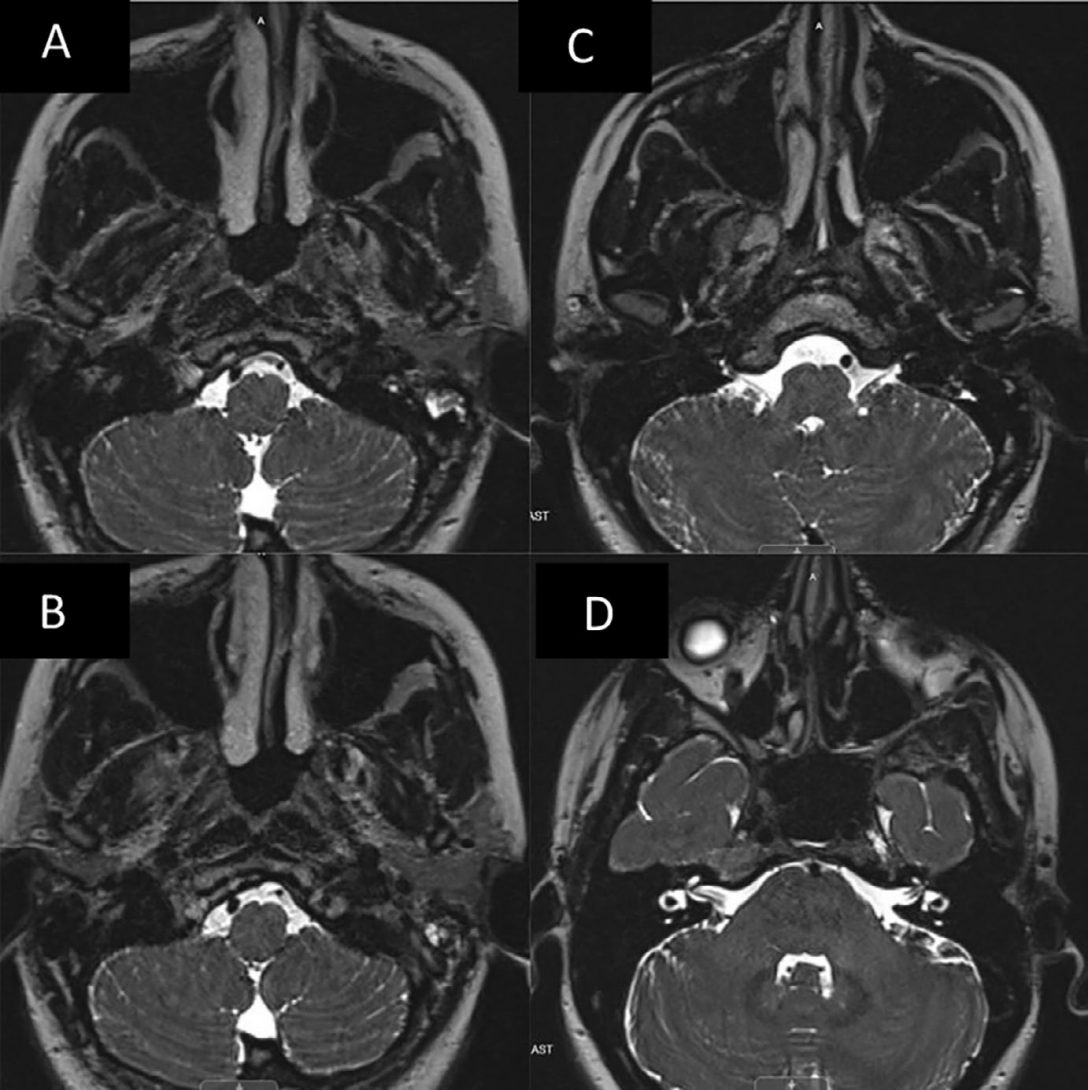
A‐D MRI brain w/o contrast, T2 weighted imaging showing (A and B, left images) increased edema of the right hypoglossal nerve compared to left as it enters the hypoglossal canal, and (C and D, right top/bottom images) cranial nerves 9–11 exiting the jugular foramen and 7–8 exiting the IAC for comparison.

At the 3‐month mark, she presented to the ED with complete resolution of her initial symptoms but noted new dysphagia and tongue weakness over the past week. She had severe atrophy on the right side of her tongue and had great difficulty moving it to the left. She had no other cranial nerve dysfunctions. Speech was fluent without dysarthria. She had dysphagia without signs of aspiration. Imaging demonstrated a healing fracture obscuring the internal opening of the right hypoglossal canal as well as perineural edema of the intracanalicular portion of the nerve (Figures 1, 2, 3, 4). The options of observation, steroids, or surgical decompression of the right hypoglossal canal were discussed with the patient, who preferred conservative management. In light of perineural edema on MRI, steroids were offered in an attempt to alleviate any nerve inflammation which resulted from compression by callus formation. She took methylprednisolone for 6 days in a tapered fashion and was advised to make appointments with ENT and SLP as well. Methylprednisolone (4 mg tablets) were administered as follows: six tablets on day 1, five tablets on day 2, four on day 3, continued in this manner until the last tablet was taken on day 6. Laryngoscopy performed by ENT confirmed no other palate deformities or swallowing malfunctions indicative of additional lower cranial nerve injuries. SLP evaluation noted modest, gradual improvement in tongue paresis. Via telehealth encounter at the 1‐year mark, the patient stated that her HNP had completely resolved 3 months after its delayed onset.

## 3 Methods

In addition to a thorough chart review for purposes of describing this case, a comprehensive literature review using the PubMed, MEDLINE, and Scopus databases was conducted. The search strategy included a combination of medical subject headings (MeSH) and keywords related to “hypoglossal nerve injury,” “occipital condyle fracture,” “delayed nerve palsy,” and “skull base trauma.” The search was restricted to articles published in English from 1980 to the present. Additional relevant articles were identified by reviewing the references of the selected studies.

Inclusion criteria were original case reports, case series, and reviews that discussed delayed hypoglossal nerve palsy following skull base trauma. Exclusion criteria included articles not available in full text, non‐traumatic causes of hypoglossal nerve injury, and studies involving pediatric populations. The selected articles were then reviewed in full, and data were extracted regarding the incidence, pathophysiology, management, and outcomes of delayed hypoglossal nerve palsy secondary to occipital condyle fractures.

## 4 Conclusion and Results

The literature review highlights cases of isolated hypoglossal nerve palsy following occipital condyle fractures (OCF), primarily resulting from motor vehicle accidents. Patients, ranging in age from 16 to 45, typically experienced a delayed onset of hypoglossal nerve palsy, with symptoms appearing anywhere from 3 days to 12 weeks post‐injury. Imaging frequently revealed fractures traversing the hypoglossal canal, sometimes accompanied by callus formation. Treatments varied, including the use of soft or rigid cervical collars, decompression surgeries, and steroids, while some cases received no intervention. Outcomes were mixed, with many patients showing no change in palsy, though a few achieved complete resolution or mild improvement.

This case illustrates the delayed fashion in which the palsy can present, and represents, to our knowledge, the only reported case of a true delayed HNP from OCF that completely resolved without surgical intervention. Moreover, it underscores the need for clinicians to be vigilant for delayed nerve palsies in skull base fractures. We present a case of delayed hypoglossal nerve palsy caused by the healing process of an occipital condyle fracture. Through a multidisciplinary approach including neurosurgery, ENT, and SLP, a complete recovery is possible from this rare secondary injury.

## 5 Discussion

Isolated hypoglossal nerve palsy is a rare complication of traumatic occipital condyle fracture, and can evade diagnosis by presenting in a delayed fashion. Atraumatic cases are even rarer, and occasionally involve cervical vertebral junction tuberculosis [2, 7, 8]. Moreover, HNPs that result as a consequence of OCFs are notoriously difficult to treat. In fact, from 1989 to 2024, only 11 cases of isolated HNP have been reported, with only three achieving complete resolution (see Table 1). Traumatic cases of OCFs more commonly result in Collet‐Sicard syndrome (unilateral palsy of the lower cranial nerves) and it has been suggested that isolated hypoglossal nerve palsies are less common because the anterior condylar canal is located near the jugular foramen, within 7 mm on the intracranial side and 3 mm on the extracranial side [1]. Vadivelu et al. were the first to demonstrate a causal relationship between decompression of the canal and resolution of neuropraxia and HNP [2]. This supports the idea that callus formation over the nerve during the healing process is responsible for the delayed presentation of the HNP.

**TABLE 1:** Literature review with previously reported cases of isolated hypoglossal nerve palsy following occipital condyle fracture.

Author/year of publication	Age/sex	Mechanism of injury	CN12 palsy onset/time to onset	Imaging finding	Intervention	CN 12 palsy outcome
Orbay et al. (1989) [9]	37 M	MVA	Delayed/12 Weeks	Left OCF traversing hypoglossal canal	Soft cervical collar	Unchanged
Wasserberg et al. (1995) [10]	39 M	MVA	Delayed/3 weeks	Left OCF with displaced spinal axis 11 mm to the right of skull base	None	Unchanged
Castling et al. (1995) [11]	21 M	MVA	Delayed/6 days	Right OCF through basiocciput	None	Unchanged
Noble et al. (1996) [3]	33 M	MVA	Delayed/not reported	NA	None	Not reported
Demisch et al. (1998) [4]	45 F	MVA	Delayed/9 weeks	Right OCF traversing hypoglossal canal with callus formation	Rigid cervical collar	Unchanged
Muthukumar (2002) [12]	32 M	MVA	Delayed/3 weeks	Right OCF	Rigid cervical collar	Mild improvement
Rué et al. (2013) [13]	19 M	MVA	Delayed/15 days	Displaced left OCF upwards and inwards with a fracture through the foramen of the left hypoglossal nerve	Cervical brace	Unchanged
Vadivelu et al. (2017) [2]	20 F	MVA	Delayed/6 weeks	Anderson and Montesano Type III OCF with bone fragment resting in the epidural space at the foramen magnum‐C1 junction	Decompression, bone fragment removal, 0‐C1 fusion	Complete resolution
Ucler et al. (2018) [6]	16 F	MVA	Delayed/3 days	Left OCF extending from the hypoglossal canal to inferior clivus	Methylprednisolone and rigid cervical collar for 2 months	Complete resolution
Dattatreya et al. (2019) [8]	44 M	MVA	Delayed/12 days	Minimally displaced right OCF	Philadelphia collar	Unreported
This Case	25 F	MVA	Delayed/12 weeks	Right OCF	Cervical thoracic orthosis for 6 weeks and steroids	Complete resolution

Abbreviations: CN, Cranial Nerve; MVA, Motor Vehicle Accident; OCF, Occipital Condyle Fracture.

Management of OCF is often conservative, and relies on the use of a rigid cervical collar for 6 weeks or longer [7, 14]. However, to our knowledge, there is no other reported evidence of complete resolution of HNP through conservative management alone. In one notable exception, Ucler et al. report the use of methylprednisolone to treat HNP 3 days following OCF [6]. However, the rapid onset and resolution of the HNP suggests that the palsy was a result of edema and not callus formation or permanent damage to the hypoglossal nerve. Our patient achieved complete resolution of HNP not only through conservative treatment, but also with the use of an interdisciplinary team of ENT and SLP.

Nonetheless, given the singular nature of the case, the findings may not generalize to a broader population or predict outcomes for all patients with similar injuries. Additionally, the conservative, non‐operative management observed here, though successful in this instance, requires further investigation to assess its broader efficacy and safety in similar cases. Larger studies or randomized controlled trials are necessary to determine the reliability and applicability of conservative treatment approaches for delayed hypoglossal nerve injuries across a diverse patient population.

## Author Contributions

**Malik S. Obeidallah:** conceptualization, formal analysis, writing – original draft. **Max Fleisher:** conceptualization, data curation, formal analysis, writing – review and editing. **Peter Harris:** writing – review and editing. **Khashayar Mozaffari:** writing – review and editing. **Michael Rosner:** supervision, validation.

## Consent

I, Malik Sameer Obeidallah, affirm that written informed consent has been collected from the patient by the authors, and proof of consent is available upon request.

## Conflicts of Interest

The authors declare no conflicts of interest.

## Data Availability

The data that support the findings of this study are available on request from the corresponding author. The data are not publicly available due to privacy or ethical restrictions.
